# Beyond Fortification: Advanced Delivery Systems and Formulation Frameworks for Next-Generation Nutraceutical Beverages

**DOI:** 10.3390/foods15213837

**Published:** 2026-10-27

**Authors:** Inamul Hasan Madar, Adhil Anver Salim, Niyaz Ali-Haneef, Punitha Muruganantham, Josvitha Dsouza, Ahmed AbuSamak, Esayed Ahmed Qeshta, Saleela Abdulla Bedrakkadu, Arvind Prabhu Thustu, Khanita Suman Chinannai, Anisa Anjum Malik

**Affiliations:** 1 Centre for Integrative Omics Data Science (CIODS), Yenepoya (Deemed to be University), Naringana, Mangaluru 575018, Karnataka, India; adhilanver@gmail.com (A.A.S.); niyazalinettana@gmail.com (N.A.-H.); punitham.biosci621@gmail.com (P.M.); ahmedabusamak20@gmail.com (A.A.); esayedqeshta@gmail.com (E.A.Q.); salilakadambu6464@gmail.com (S.A.B.); arvindprabhu24@gmail.com (A.P.T.); 2 Womy Foods and Nutraceuticals Pvt Ltd., Yenepoya Technology Incubation (YTI), Yenepoya (Deemed to be University), Naringana, Mangaluru 575018, Karnataka, India; sumankhanita6@gmail.com; 3 Multiomics and Precision Medicine Lab (MPML), Yenepoya Research Centre, YRC, Yenepoya (Deemed to be University), Naringana, Mangaluru 575018, Karnataka, India; dsouzajosvitha@gmail.com; 4 eDOmics Academy Pvt Ltd., Yenepoya Technology Incubation (YTI), Yenepoya (Deemed to be University), Naringana, Mangaluru 575018, Karnataka, India; 5 Department of Food Science and Nutrition, The Yenepoya Institute of Arts, Science, Commerce and Management (YIASCM), Yenepoya (Deemed to be University), Kulur Campus, Bangra Kulur, Mangaluru 575013, Karnataka, India; anisaanjummalik@yenepoya.edu.in

**Keywords:** nutraceutical beverages, nanoencapsulation, bioaccessibility, multi-criteria decision analysis, gut-on-a-chip

## Abstract

Functional beverages are increasingly used as a medium for targeted nutritional intervention, but liquid matrices present limitations that free bioactives frequently fail to overcome, such as precipitation, oxidation, and degradation during storage and gastrointestinal transit. This review explores the delivery systems that overcome these limitations in acidic, low-viscosity beverages, including biopolymer nanocarriers, solid-particle-stabilized (Pickering) emulsions, nanoemulsions, co-encapsulation systems and fermentation-derived exopolysaccharides. Beyond simple retention, we propose bioaccessibility (speciated) and sensory acceptability (clarity, mouthfeel, off-notes) as co-primary evaluation endpoints. The compatibility, stability, processing resilience (pasteurization, ultra-high temperature (UHT), high-pressure processing (HPP), pulsed electric field (PEF)), scalability and cost are combined in a comparative decision-support framework, and the sustainability assessment warns against assuming environmental benefits based on bio-based origin alone. Regulatory analysis compares the EU’s statutory nanomaterial framework with functional pathways in the US and UK, framing gastrointestinal dissolution screening as a pragmatic filter rather than a universal barrier. Lastly, we describe novel validation tools, gut-on-a-chip and organoid models combined with AI-powered multi-omics, not as replacements for human studies but as tools to accelerate formulation prioritization. The review proposes a matrix-aware framework for evaluating beverage delivery as a decision-governed translation problem that encompasses efficacy, acceptability, sustainability, and regulatory viability.

## 1. Introduction

The functional beverage market has grown from being just a fortified drink to a targeted health delivery system, as consumers have moved away from drinking fluids for hydration and are seeking evidence-based nutritional interventions [1,2,3]. The main challenge in this area is a stability-bioavailability paradox: the compounds of greatest value to deliver in beverages are frequently the ones that are least stable in their free form in liquid matrices [4,5,6]. The physiological value of direct fortification is often lost due to precipitation, oxidation or degradation during gastrointestinal digestion [7,8]. To overcome this, current research has shifted its focus from simple addition to encapsulation, colloidal delivery, and fermentation-based systems that modulate release, transport, and sensory behavior [4,9,10]. The objective of this review is to propose an integrated, matrix-aware translation framework to assess advanced beverage delivery systems throughout the formulation process, from delivery-system architecture and processing resilience to speciated bioaccessibility, sensory acceptability, life-cycle sustainability, and regulatory compliance.

### 1.1. The Functional Beverage Paradigm: Consumer Trends and Health Demands

Beyond vitamin fortification, consumers are now seeking probiotics, antioxidants, and specialized nutraceuticals in their daily beverages, reflecting a growing trend toward targeted wellness and preventive health measures [1,11,12,13]. But beverages are not inert carriers; the aqueous, acidic, and low-viscosity matrices of beverages determine the plausibility of health claims. Each of the major classes of bioactives to be delivered has its own unique physicochemical vulnerabilities that cannot be addressed by a single carrier system. These are the main formulation constraints and the specific molecular targets that any potential delivery architecture must address, as summarized in Table 1.

### 1.2. The Stability-Bioavailability Paradox in Liquid Matrices

The central technical challenge in beverage formulation is the stability–bioavailability paradox: bioactives of highest nutritional value are usually the least stable in aqueous, low-viscosity matrices [4,6,19]. Crucially, analytical retention in the container does not guarantee physiological availability, as liquid matrices lack the protective lipid environments of solid meals [7,21]. In water-based and fat-free drinks, oral bioavailability is constrained by rapid gastric transit and a lack of digestible co-lipids, causing released lipophilic bioactives to precipitate into unabsorbable crystals before intestinal mixed micelles can form [7,9,22]. Consequently, digestive fate is highly influenced by matrix-dependent factors [7,20]: dairy matrices substantially enhance carotenoid bioaccessibility because digestible milk fat globules supply the lipidic core required for mixed-micelle assembly [7,22], whereas in water-based juices, the absence of co-lipids leads to premature precipitation [22,23]; conversely, complex botanical matrices like coffee facilitate resveratrol uptake through co-solute solubilization while paradoxically reducing β-carotene bioaccessibility (to <0.25%) by disrupting micellar organization [23]. Furthermore, biological transport barriers diverge across bioactive classes [14,20]: lipophilic compounds (carotenoids, curcumin) require micellar incorporation for enterocyte uptake via passive diffusion and scavenger receptors (SR-BI/CD36) [6,17], whereas hydrophilic polyphenols face poor paracellular tight-junction permeability, phase II conjugation, and active luminal extrusion by apical P-glycoprotein (P-gp) efflux pumps [14].

Because beverage delivery is strictly governed by digestion-stage matrix interactions, encapsulation strategies developed for solid foods cannot be directly transferred to liquid formats [18]. However, resolving these stability and bioaccessibility problems creates a secondary formulation conflict: conventional stabilization strategies, such as dense biopolymer shells or thickened interfacial networks, directly compromise the optical clarity, low viscosity, and refreshing mouthfeel that consumers demand in liquid beverages [2]. This then creates a three-way optimization problem: maximizing bioaccessibility while simultaneously preserving physical stability and sensory palatability [18]. Navigating these parameters requires moving beyond simple payload protection toward a matrix-aware framework, in which delivery architectures are specifically harmonized with the beverage matrix to withstand processing and storage while facilitating digestive release and mucosal permeation [2,7].

### 1.3. Review Scope and Methodology

This article is a structured critical narrative review and not a formal meta-analysis. Due to the high level of heterogeneity in the matrices, carriers, in vitro digestion methods, sensory methods, and regulatory approaches, a quantitative meta-analysis was not possible or appropriate.

Literature searches were conducted across PubMed, Scopus (Elsevier), and ScienceDirect (Elsevier), covering publications from 2009 through 2026 (with >98% of retained citations originating between 2015 and 2026). Searches combined encapsulation and delivery terminology (“nanoencapsulation” OR “nanoemulsion” OR “liposome” OR “Pickering emulsion” OR “exopolysaccharide”) with beverage-matrix descriptors (“functional beverage” OR “fruit juice” OR “acidic beverage” OR “liquid matrix”).

In addition, primary regulatory and guidance documents were retrieved from EUR-Lex (European Commission recommendations and regulations), the EFSA Journal (European Food Safety Authority guidance), and the U.S. Food and Drug Administration (FDA) guidance registry.

Inclusion criteria include: (i) studies evaluating delivery systems specifically within acidic, low-viscosity liquid matrices; (ii) formulations tested against commercial thermal or non-thermal processing conditions (pasteurization, UHT, HPP, PEF); (iii) investigations reporting speciated bioaccessibility, physical stability, or sensory/organoleptic endpoints; and (iv) authoritative regulatory and guidance documents from the European Commission, EFSA, and the US FDA. Studies focusing exclusively on solid food matrices, non-food/pharmaceutical carrier vehicles, or lacking matrix-specific stability data were excluded. Ultimately, 225 peer-reviewed papers, key methodological standards, and regulatory documents were evaluated and synthesized into this review. To keep the literature evidence and the author’s interpretation separate, specific formulation values and experimental results are referred to their original sources, while the conceptual frameworks, schematic figures and comparative matrix are qualitative syntheses that contextualize the results.

### 1.4. Literature Context and Proposed Framework

Recent reviews have examined the various systems used for the encapsulation of functional foods and beverages, offering useful information on the different types of food systems for delivery [3,4,5,6,10,17,18,19]. The present review builds on these contributions and concentrates specifically on the multi-dimensional translation interface in liquid beverage matrices.

For example, Shishir et al. [4] gave an extensive review of the micro- and nano-encapsulation methods in dairy and non-dairy beverages, but their review was limited to payload retention and not to sensory detection limits or post-digestion speciation. While Tarahi et al. [5] gave a detailed review of plant-based delivery vehicles and their release behaviors, our review builds on this mechanistic understanding by assessing the performance of these biopolymers under commercial beverage processing conditions (UHT, HPP, PEF). Likewise, although Liu et al. [10] investigated functional food nanoencapsulation in a wide range of formats, the current study is limited to the specific colloidal stability, optical clarity and low pH aspects of liquid beverages. While Choi and McClements [17] and Aditya et al. [18] detailed the fundamental colloidal physics of nanocarriers, our review translates these principles to commercial beverage formulation, processing, and regulatory limitations. Lastly, although market overviews, like that of Hilton [3], provide information on consumer trends and industry growth, this review aims to link these commercial drivers to a structured decision-support framework for formulation development.

To bridge these domains, this review proposes an integrated matrix-aware translation framework structured around the following dimensions:

(i) a matrix-aware review of the colloidal and physical constraints specific to acidic (pH ≤ 4.5), low-viscosity beverages;
(ii) the proposed consideration of compound-resolved (speciated) bioaccessibility along with sensory acceptability (clarity, mouthfeel, and off-note masking) as complementary evaluation endpoints;
(iii) an overview connecting carrier stability under thermal (pasteurization, UHT) and non-thermal (HPP, PEF) processing with industrial scalability and formulation cost considerations;
(iv) a comparative analysis of regulatory pathways and evidentiary considerations across the European Union, the United States, and the United Kingdom; and
(v) a forward-looking perspective on how emerging microfluidic gut-on-a-chip, organoid, and computational approaches may help in formulation prioritization to complement, rather than replace, human studies.

## 2. Carrier Architectures for Beverage Bioactives

### 2.1. Nano-Encapsulation Platforms: Liposomes, Niosomes, and Cyclodextrin Complexes

When formulating liquid beverages to include bioactives, selecting between vesicular carriers (liposomes, niosomes) and supramolecular inclusion complexes (cyclodextrins) generally comes down to balancing two intersecting considerations: the physical demands of the beverage matrix (such as optical clarity, pH, and pasteurization conditions) and the targeted biological fate of the bioactive (including taste masking, gastric transit survival, and intestinal absorption) [16,24,25]. Rather than acting as universal or interchangeable substitutes, these systems operate through fundamentally different physical mechanisms. Consequently, each platform tends to align with specific beverage formats while introducing its own operational and digestive trade-offs [26,27,28].

Liposomes are self-assembled closed bilayers composed of amphiphilic phospholipids, entrapping an aqueous core for hydrophilic actives and lipid lamellae for lipophilic payloads [25,29,30]. Because their colloidal size (>100 nm) causes light scattering and turbidity, empirical beverage applications have focused mainly on naturally opaque matrices, such as fortified dairy drinks and plant-based milks [25,29,30]. In acidic or pasteurized systems (pH ≤ 4.5), unmodified ester-linked phospholipids undergo acid hydrolysis and lipid peroxidation, which can be mitigated by electrostatic deposition of GRAS biopolymers (e.g., pectin, alginate, whey protein) or hydrogel embedding [26,29,31,32]. Biologically, liposomal uptake depends on intestinal lipid digestion, requiring pancreatic lipolysis and bile-salt micellarization for epithelial absorption [25,29]. While thick biopolymer shells can sterically hinder lipase access and retard micellization [26,32], calibrated coatings preserve digestive efficacy: pectin-coated lyophilized liposomes co-encapsulating vitamins B12 and D3 maintained physical stability and sensory palatability in yogurt while sustaining intestinal bioaccessibility during INFOGEST digestion [29]. Therefore, unmodified vesicles are best suited to refrigerated, near-neutral dairy products, whereas coated vesicles must still be validated in real beverage matrices rather than simplified buffers [16].

Niosomes have a vesicular structure similar to liposomes, but self-assemble from non-ionic surfactants and sterols without hydrolyzable ester bonds [33,34,35]. This chemical property is expected to provide better resistance to low pH and thermal processing, offering a potential alternative platform for acid-stressed or pasteurized matrices [33,34,35]. The rigidity of the vesicles can be optimized to resist acid-induced aggregation by adjusting the hydrophilic–lipophilic balance (HLB) and sterol ratio [33,34,35]. This stability has shown promising results in model beverage systems: phytosterol-stabilized niosomes exhibited good retention of quercetin during thermal pasteurization and 30-day refrigerated storage in an orange juice model [33]. Mechanistically, the acid- and enzyme-resistant bilayer implies that labile payloads like polyphenols and peptides should be protected from gastric degradation; however, direct empirical evidence in beverage matrices remains limited [33,34], though comparative in vitro studies indicate that niosomes release their payload more sustainably than liposomes under physiological buffer conditions [36]. Furthermore, in beverage models fortified with the antihypertensive tripeptide Ile-Pro-Pro, niosomal formulations demonstrated extended storage stability and favorable sensory palatability, despite having larger vesicle sizes and higher polydispersity than liposomes [36]. That said, the number of approved non-ionic surfactants for commercial beverages is much smaller than in pharmaceutical delivery, and surfactant residues must be carefully optimized organoleptically to prevent bitterness or soapy off-notes [35,37].

Cyclodextrins (CDs) work through a distinct supramolecular host–guest mechanism rather than colloidal compartmentalization [24,28,38,39]. CDs are structurally distinct, featuring a hydrophobic cavity and a hydrophilic exterior, and form true molecular solutions that scatter no visible light, making them ideal for applications where optical clarity is mandatory (such as ready-to-drink teas, clear functional waters, and sports beverages), whereas colloidal vesicles inherently produce turbidity [28,38,39]. In the oral stage, host–guest entrapment can physically separate hydrophobic bitter or astringent moieties (e.g., polyphenols or caffeine) from lingual taste receptors, helping to reduce the perception of off-notes while maintaining optical clarity in aqueous solution [28,38,39]. In the gastrointestinal tract, CDs keep lipophilic guests in dynamic molecular dispersion, potentially enhancing luminal dissolution and passive mucosal permeation without the need for enzymatic lipolysis or bile-salt micellarization [28,38,39]. Standalone CDs, however, cannot encapsulate hydrophilic compounds, have low stoichiometric loading capacity (typically 1:1 or 1:2 molar complexes), and are susceptible to competitive displacement of guest molecules by beverage volatiles [27,38]. To address the low loading efficiency of single carriers, hybrid cyclodextrin-in-liposome architectures have been investigated in model delivery systems. In such systems, pre-complexing poorly soluble hydrophobic guests with CDs before vesicular entrapment has been shown to improve apparent aqueous solubility (up to 11-fold in model studies) and significantly improve core loading [27,40]. However, because vesicular structures inherently scatter light and induce turbidity, such hybrid architectures are unsuited for transparent beverages, remaining primarily a conceptual proof-of-concept for opaque or emulsion-based matrices that requires substantial adaptation for food products [16,27,40]. In short, these platforms appear to occupy distinct formulation niches rather than serving as universal solutions: vesicular carriers show the greatest utility for multi-compartment delivery in opaque or pasteurized drinks, whereas cyclodextrins are particularly advantageous for taste masking and dissolution where optical clarity is essential [4,16,18].

### 2.2. Colloidal Systems: Pickering Emulsions and Nanoemulsions for Optical Clarity

Pickering emulsions and nanoemulsions represent the two most relevant colloidal strategies for delivering lipophilic ingredients in clear beverages, yet they achieve stability and optical performance through fundamentally different physical mechanisms [41,42]. Pickering emulsions are stabilized by solid particles adsorbed at the oil–water interface, forming a dense mechanical barrier that suppresses coalescence and retards Ostwald ripening more effectively than many conventional surfactant systems [43,44]. Their primary advantage for next-generation beverages lies in the utilization of food-grade particles, such as starch, cellulose, chitosan, and composite biopolymers, which aligns with clean-label formulation goals while offering superior robustness against pH, temperature, and ionic strength fluctuations compared to molecular surfactants [42,44,45]. Nanocellulose is particularly promising in this regard, as its high aspect ratio allows it to form three-dimensional networks between droplets, stabilizing oil-in-water systems relevant to drinkable products [42,44,46]. Furthermore, Pickering water-in-oil-in-water (W/O/W) multiple emulsions offer a viable route to beverage fat reduction, replacing bulk fat with internalized water phases while still protecting sensitive bioactives [41,47]. However, achieving optical clarity remains a critical limitation for Pickering systems. True transparency requires not only sub-micron particle sizes and favorable wettability, but critically, a low-viscosity, liquid-like behavior, whereas many robust Pickering formulations rely on a gel-like particle network that renders the beverage opaque [48,49].

In contrast, nanoemulsions are the more established platform for optical clarity. Because droplet sizes below approximately 200 nm scatter minimal light, they enable the formulation of transparent or translucent fortified waters and juices [41,50]. Rather than relying on a mechanical interfacial barrier, nanoemulsions suppress droplet growth by drastically reducing the Laplace pressure that drives Ostwald ripening. Despite this kinetic stability, nanoemulsions remain thermodynamically unstable and can still undergo droplet growth over extended storage, a risk often mitigated by the high energy inputs, such as high-pressure homogenization or ultrasonication, required for their production [50,51].

The selection between these two systems ultimately hinges on a complex sensory trade-off. Pickering emulsions can intentionally increase viscosity and mimic creamy, fat-like textures, a valuable tool for mouthfeel engineering in low-fat formulations, but inherently at odds with the thin, refreshing mouthfeel expected of clear drinks [43,52]. Nanoemulsions generally avoid grittiness and maintain a low-viscosity profile, yet their exceptionally high interfacial area and associated emulsifier loads can still subtly alter oral lubrication and mouthfeel [42]. Consequently, nanoemulsions currently provide the most practical and reliable route to transparent beverage fortification, while Pickering emulsions are reserved for formulations where clean-label stabilization, targeted fat reduction, or engineered texture are prioritized over absolute visual clarity [51,53].

### 2.3. Co-Encapsulation Strategies for Synergistic Phytochemical Delivery

Co-encapsulation is most rational when two actives are mechanistically complementary and formulation-limited when administered separately. Simple blending can often result in the loss of bioactive value due to the poor aqueous solubility, chemical instability, and rapid metabolism of many phytochemicals, as well as low oral bioavailability. To overcome this, co-delivery systems have been developed to shield multiple actives in a single carrier, control the release of actives, and provide synergistic effects at lower effective loads [54,55,56]. Other combinations such as curcumin with resveratrol, quercetin, or EGCG also exhibit synergistic or complementary bioactivity, with evidence supporting the health benefits being more robust in preclinical studies than in actual foods or human trials [57,58].

From a mechanistic point of view, three co-encapsulation approaches are most relevant to beverages. For hydrophilic–hydrophobic pairs or probiotic–phenolic systems, the actives should be incompatible and located in different parts of the same carrier, which is called compartmentalization [56,59,60]. In water-in-oil-in-water (W/O/W) emulsions, curcumin and EGCG were encapsulated in two different phases, and the protein fibril–cellulose stabilization enhanced the heat and salt resistance and increased the bioaccessibility of curcumin to 67.8% and EGCG to 68.9% [59]. Sequential release systems are systems where one active is protected via the stomach, and the other is released later in the intestine or colon. Although these models are not well described in liquid beverages, they set a high standard for spatial release control in advanced oral dosage forms. For instance, in a solid-in-oil-in-water (S/O/W) multilayer emulsion, gastric release was maintained at less than 16.5%, intestinal release of curcumin was 67.3%, colonic release of fucoxanthin was 60.3%, and the bioavailability of curcumin was enhanced 6.4-fold in vivo [61]. The proof-of-concept is mechanistic, and while translating these multi-layered, highly structured architectures into low-viscosity, shelf-stable drinks is a big formulation challenge, it offers a clear target for future beverage-specific colloidal design.

For beverage applications, a case-study framing is appropriate, as it demonstrates that co-encapsulation can enhance the function and product stability. The zein/carboxymethyl chitosan nanoparticles co-loaded with curcumin and resveratrol yielded ~201.6 nm particles with 72.9% and 78.2% encapsulation efficiencies, respectively, and exhibited better gastrointestinal stability and bioaccessibility than free compounds [62]. A co-encapsulation system of zein and BSA was used to improve the encapsulation efficiency and shelf stability of resveratrol, where the two components were located in different regions of the nanoparticles and released at different rates [63]. When both drugs were co-loaded into the same liposome, the resulting particles were smaller and more uniform, and the antioxidant activity was greater than that of single drug loading; curcumin was found to be in the bilayer core, while resveratrol was found to be near the polar head groups, which seems to stabilize the carrier itself [64]. The relevance of beverages is not theoretical: hyalurosome co-encapsulation of curcumin and resveratrol was developed specifically for juice and yoghurt, and was found to offer nano-scale dispersibility, gastrointestinal stability, and increased radical-scavenging activity compared to single compounds or conventional liposomes [65].

Although these mechanistic advances have been achieved, there is a significant ‘Valley of Death’ between co-encapsulation at the lab scale and commercial production of beverages. Simple spray-dried microcapsules are well established in the industry for dry premixes, while more complex microcapsules such as W/O/W double emulsions, sequential multilayer emulsions and liposomal hybrids are still at the bench-scale or proof-of-concept stage. This translation gap remains due to the high complexity of scale-up, charge screening, payload leakage and oxidation penalties associated with multi-component fabrication that are significantly exacerbated in acidic, shelf-stable liquid matrices. Thus, cost and process simplicity are as important as synergistic bioactivity, and future advances will rely on carriers that can be fabricated without undue formulation burden, using food-grade materials, and be scalable [10,56,58].

### 2.4. Fermentation as a Dual-Purpose Delivery Mechanism

Unlike conventional delivery systems that require pre-isolated active compounds and externally added carriers, fermentation operates as a biological platform that simultaneously bioactivates the nutritional payload and synthesizes the protective carrier matrix in situ [66,67]. This dual-purpose strategy applies across diverse functional drink categories, including fermented dairy and plant-based milks (kefir, drinkable yogurts, and fermented soy), fermented botanical infusions (kombucha), and lacto-fermented fruit and vegetable juices (e.g., blueberry and pomegranate), where microbial metabolism coordinates payload transformation with in situ matrix structuring [4,67,68,69]. Rather than serving merely as a preservation technique, fermentation alters the physical and physiological properties of the beverage through two interconnected pathways: the enzymatic cleavage and biotransformation of complex plant phytochemicals into smaller, more bioavailable metabolites (Section 2.4.1) [68,70], and the simultaneous secretion of functional exopolysaccharides that provide clean-label rheological stability while offering potential as gut-directed protective matrices (Section 2.4.2) [69,71].

#### 2.4.1. In-Situ Bioconversion to Highly Bioavailable Metabolites

In-situ bioconversion places the fermentation process as the main delivery intervention. Esterases, decarboxylases, reductases and glycosidases are enzymes produced by microbes, especially lactic acid bacteria (LAB), that break bound phenolics from plant cell matrices and transform hydroxycinnamic acids, hydroxybenzoic acids and flavonoid glycosides into downstream metabolites that have different solubility and bioactivity [68,72]. This is a paradigm change from adding a pre-encapsulated ingredient to designing the food matrix to bioactivate the active in situ during processing [68,70,72].

This mechanism is supported by a representative food system. The activity of LAB strains, such as *Pediococcus pentosaceus*, which have high levels of β-glucosidase and ferulic acid esterase, resulted in the release of bound phenolics into free phenolics, with free p-coumaric acid increasing by 30–3000% and free ferulic acid increasing by 203–359% in fermented bamboo shoots [73]. Likewise, the phenolic biotransformation, antioxidant capacity, and phenolic bioavailability of blueberry systems were significantly improved by LAB fermentation, which was observed during subsequent digestion [74]. Similar improvements have been observed with various substrates, such as orange peel, kiwifruit, and wheatgrass juice, and fermentation has been shown to be a reliable method for converting phytochemicals into more bioavailable forms [72,73].

Although these achievements have been made, the main drawback of in-situ bioconversion is its strong reliance on the microbial strain, substrate chemistry, and matrix complexity. In real food environments, the expression of microbial genes is often different from what has been predicted in vitro due to the dynamic nature of the nutrient landscape [68]. In addition, the baseline bioavailability is highly dependent on the plant matrix, meaning that the best biotransformation strategy for LABs should be optimized on a case-by-case basis rather than as universal protocols [70]. Therefore, fermentation cannot be considered a system for targeted delivery, but rather a powerful pre-processing step to transform poorly accessible phytochemicals into highly bioavailable forms and thus minimize the formulation challenge for downstream encapsulation technologies [70,73].

#### 2.4.2. Microbial-Derived Exopolysaccharides as Natural Encapsulants

Microbial EPS are natural, multifunctional encapsulants produced during fermentation. In the literature, LAB derived EPS are known as biothickeners and rheology modifiers in both dairy and non-dairy drinks, which improve the consistency, physical stability, emulsification and gelation of the drinks [66,75,76]. Synthesized by microorganisms that are edible, EPS are compatible with the clean label formulation goals, which means that the use of synthetic hydrocolloids is reduced and the by-products of the fermentation process are themselves valorized [69,73,77].

In addition to the techno-functional role, EPS play a critical gut-directed role. Prebiotic effects have been shown for EPS produced by different strains, such as *Schleiferilactobacillus harbinensis*, *Leuconostoc mesenteroides* and *Lactiplantibacillus plantarum*, and ex vivo fecal fermentation models have confirmed that EPS with lower molecular weight are produced, and that the production of butyrate or propionate is increased [78,79,80]. In an ex vivo colon model, a fermented soy beverage with EPS showed greater alpha diversity of the microbiota and higher levels of butyrate and propionate than the base beverage, in a direct beverage application [81].

But it is too early to say that EPS is really a “controlled-release carrier” in the pharmacokinetic sense. The existing evidence base is overwhelmingly techno-functional stabilization and prebiotic fermentation, with no quantitative data on the site-specific in vivo release of co-loaded exogenous actives [69,82]. The most compelling indirect evidence is that EPS are resistant to gastric conditions and then broken down in the colon by the microbiota, which is a type of biologically triggered release, not an engineered control [69,79]. In addition, the low production yields and the highly structure-dependent fermentability of EPS, coupled with the inter-host variability in microbiota which may result in non-responder phenotypes [69,76,83], limit the widespread use of these microorganisms. Thus, EPS have already been proven as natural stabilisers and prebiotic matrices, and are a promising research area that needs to be investigated for precise and controlled release in beverages, detailed structure–function relationships, and scalable production.

## 3. Bridging Efficacy and Acceptability: Bioaccessibility and Sensory Trade-Offs

The architectural strategies described in the previous sections, such as hybrid nanocarriers, Pickering emulsions, co-encapsulation and precision fermentation, are all designed to ensure that bioactives remain protected in the harsh physicochemical environment of liquid matrices, but their translation from the laboratory to the consumer’s glass relies on two key dimensions: rigorous physiological validation and uncompromised sensory quality [1,4]. It is not enough to show that a carrier is able to protect a payload during shelf storage; it is necessary to prove that the system improves the actual physiological fate of the bioactive, without causing unacceptable organoleptic defects [7,9]. This section thus connects formulation design and real-life application. It first considers the analytical standardization challenge, moving away from bulk compositional metrics to the concept of speciated bioaccessibility, followed by a discussion of the sensory challenges and trade-offs that are introduced by advanced delivery architectures, such as turbidity, mouthfeel and off-notes, and the strategies needed to overcome these challenges [2,4].

### 3.1. The Analytical Standardization Problem: Total Phenolics Versus Speciated Bioaccessibility

Bulk colorimetric assays are still commonly used in the study of nutraceutical beverages. However, they cannot be used as independent indicators of functional composition as they are only an indirect measure of the total reducing capacity of the mixture and not of the individual phenolics. The Folin-Ciocalteu response also varies based on the structure of the phenol, and thus cannot be used to determine the behavior of different molecular forms within a formulation or digestion process [84,85].

This limitation is important because food and beverage matrices may interfere with the total phenolic results due to chemical interference and matrix effects [84,86,87]. Proteins are also known to be a source of Folin-Ciocalteu reactivity [88], and heat processed matrices may also overestimate phenolic content if Maillard-derived melanoidins contribute to the assay response [89]. The interference by simple sugars should be mentioned with caution, however, as one validated Folin-C method reported no positive interference from glucose, fructose, and sucrose under the conditions tested [90].

Total retention is thus not a complete endpoint for beverage delivery systems. In the gastrointestinal digestion, total phenolics may not change or even increase, while some compounds may be hydrolyzed, degraded, or converted into other species, as highlighted in the digestion models used in studies [91,92,93]. In digested cocoa beverages, for example, apparent total phenolic bioaccessibility was 129.71% due to enzyme release of protein-bound complexes and peptide interferents; however, compound-specific UHPLC-HRMS revealed that 8 of the 10 parent phenolics had degraded, leaving only 2 detectable [93,94].

To resolve this disparity between bulk assay and actual molecular fate, a more sophisticated analytical approach would be to combine bulk screening assays with compound resolved chromatography. When the objective is to quantify individual phenolics, to evaluate processing effects, or to monitor the changes in their composition that occur during digestion, chromatographic methods are required [87,95,96]. High-performance liquid chromatography–tandem mass spectrometry (HPLC-MS/MS) can overcome the problem of endogenous matrix overlap that prevents the detection of compounds by less selective detectors in digested samples [95].

Along with chromatographic speciation, standardized in vitro digestion is also a key element of comparability. INFOGEST 2.0 offers a unified approach to gastrointestinal digestion simulation and has significantly enhanced consistency among phenolic bioaccessibility studies [93,94]. Despite this, there is still no complete agreement in the literature regarding sample preparation, reporting format and bioaccessibility calculations, which still restricts the possibility of cross-study comparison [94,97].

Therefore, the next generation of beverage evaluation should shift from total retention to speciated bioaccessibility, identifying the different forms of phenolics in the beverage, monitoring their transformation during digestion, and quantifying the amount of phenolics that are solubilized in the intestinally available phase [98]. If the goal is physiological relevance, not only compositional screening, digestion outputs can be extended into intestinal transport models such as Caco-2 monolayers to estimate which released phenolics are likely to cross the epithelial barrier.

**Figure 1:**
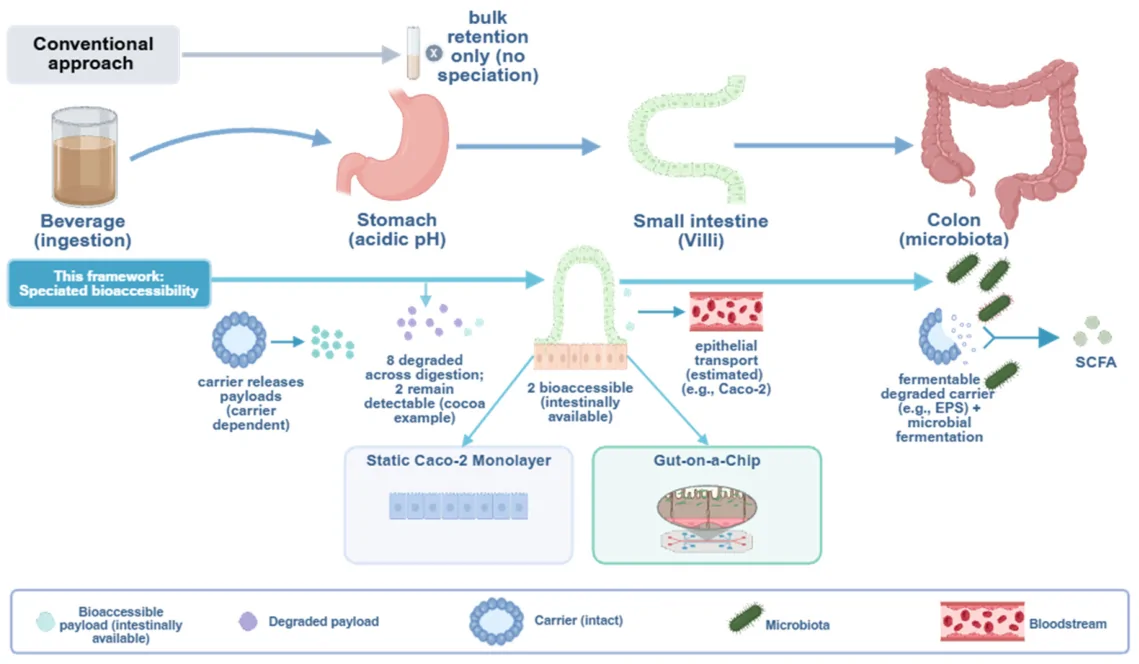
From bulk retention to digestive fate: reframing beverage bioaccessibility. The upper track shows the conventional bulk/total-retention readout; the lower track follows one speciated payload through ingestion, gastric transit, intestinal bioaccessibility and colonic fermentation. Species counts (8 degraded/2 remain detectable and bioaccessible in the intestinal phase) mirror the cocoa intestinal example in Section 3.1 (two of ten phenolics detectable after digestion; [93,94]) and are schematic, not cross-study measurements; epithelial transport is shown as an estimated step without counts [99]. The static Caco-2 monolayer (no flow; lacks shear and mucus) is contrasted with gut-on-a-chip platforms validated in liquid food and beverage-relevant matrices (Section 6.1), which accelerate formulation prioritization but do not replace human studies.

This reframing, from a single bulk value to the species-resolved digestive fate of the payload, and from static monolayers to dynamic liquid-matrix models is summarised in Figure 1.

### 3.2. Achieving a Balance Between Bioavailability, Turbidity, Mouthfeel and Off-Notes

Beyond shelf retention, the delivery value of a functional beverage is ultimately judged by its speciated bioaccessibility [4,7], which must be negotiated against a three-way tension between optical clarity, oral palatability, and intestinal release [4,17]. Optically, water-clear RTD products tend to require droplets well below visible wavelengths (roughly under 50–100 nm, within the Rayleigh regime), whereas diameters approaching 100–200 nm increase Mie scattering and typically leave the product translucent at best [17,50]. The surfactant-to-oil ratios needed to reach this size range, however, may carry a sensory cost: unadsorbed emulsifiers have been implicated in depleted salivary lubrication and elevated boundary friction, which consumers may perceive as drying or astringent mouthfeel [52] (tribological endpoints are discussed in Section 3.2.2). Physiologically, the same dense interfaces that help shield bitter moieties from lingual taste receptors are also thought to slow duodenal lipase–colipase adsorption, potentially delaying lipid digestion and limiting micellar bioaccessibility [4,17]. Balancing these competing demands, rather than maximizing any one of them, is the formulation problem taken up in Section 3.2.1 and Section 3.2.2.

#### 3.2.1. Case Studies in Sensory—Functional Trade-Offs

Nanoemulsions are the most well-known method to obtain transparent or translucent beverage fortification, as they scatter less light and also enhance the dispersibility and delivery of lipophilic compounds [50,100,101,102]. This optical benefit is usually linked to droplets smaller than approximately 200 nm, where the system becomes more homogeneous and less likely to flocculate in the visible range [50]. Even though the initial appearance of the nanoemulsions is satisfactory, the droplets may grow in size during storage, resulting in creaming, sedimentation, coalescence, or Ostwald ripening, which can compromise the clarity of the product [100,103]. Their practical limitation in beverages is not just whether they can enhance bioaccessibility, but whether they can meet the requirement of increased payload, formulation complexity, or longer shelf-life without making droplet characteristics fall out of the optical window of a visibly clear product [102,103].

Pickering emulsions have a different balance between mouthfeel and stability due to the presence of solid particles at the oil–water interface, which provide a dense mechanical barrier that enhances resistance to coalescence and environmental stress [53,104]. They are also attractive in the food and beverage sector due to the use of plant- and biopolymer-based particles, which can aid in clean-label positioning and improve texture and functional ingredient retention [52,53]. Pickering emulsion gels can mimic the sensory properties of fat and enhance the mouthfeel of reduced-fat systems, such as low-oil emulsion gels and pomelo-fibre systems developed as fat mimetics [52,105,106]. However, the same structuring that enhances creaminess can go too far: adding more crystals or particles can lead to decreased lubricity, increased brittleness, aggregation, and a gel-like or intrusive mouthfeel in the beverage [106,107].

Cyclodextrins can be used for off-notes versus protection, as they are able to form inclusion complexes with hydrophobic guests and are commonly used in beverages to stabilize volatiles, enhance shelf life, and mask bitterness and undesirable odors and flavors [108]. Their masking effect is also confirmed in application-oriented studies, such as EGCG beverages, where the use of nanocomplexes containing β-cyclodextrin led to a decrease in the perception of bitterness and astringency [109]. The performance of cyclodextrin is not absolute, however, as guest molecules compete for the β-cyclodextrin cavity, competitive release can be achieved by other compounds, concentration changes, or storage conditions [110,111,112]. In addition, Mai et al. [112] demonstrated that citrus off-flavor compounds were decreased by the use of β-cyclodextrin. However, the encapsulation efficiency was influenced by pH, temperature, acids and storage conditions, indicating that masking performance may be compromised in complex beverage matrices [112].

Lipid-based carriers address another aspect of the flavor issue by protecting sensitive actives from the aqueous phase, but they also create a potential for oxidation to produce new off-notes. In emulsions, lipid oxidation is a significant degradation pathway, and in nanoemulsions, it is sometimes enhanced by the increased interfacial area, which leads to more contact between the oil phase reactants and water-soluble pro-oxidants [103,104]. This is directly related to sensory quality, as volatile aldehydes like pentanal and hexanal are known markers of oxidation-induced flavor spoilage [113]. However, the oxidation results are not invariably poorer in all nanoscale systems: Maher et al. [113] reported that spray-dried nanoemulsions had lower levels of pentanal and hexanal than conventional emulsions, and Pascual-Silva et al. [114] demonstrated that antioxidant-loaded liposomes exhibited better physical and oxidative stability and lower hydroperoxide and thiobarbituric acid reactive substances (TBARS) accumulation.

#### 3.2.2. Formulation Strategies for Mitigation

These formulation trade-offs are best managed by considering sensory aspects early in carrier design alongside bioaccessibility [4,7,18,103]. This approach benefits from distinguishing instrumental screening from human sensory evaluation. Instrumental tools, such as electronic tongues for bitterness profiling [110] and oral tribology for measuring boundary lubrication [52,106], offer rapid, objective pre-screening, but cannot capture cross-modal perception or hedonic liking [2,109]. Therefore, sensory evaluation should be appropriately differentiated: trained descriptive panels (e.g., QDA) are most suitable for formulation refinement and tracking specific attribute intensities (such as lingering astringency or oxidation off-notes) [109], whereas consumer testing remains essential in later stages to confirm overall palatability, refreshing character, and product acceptance [2,29,36].

The second priority is the stability design that is compatible with the process. The droplet size distribution and preparation route are important factors to consider when preparing nanoemulsions as the stability of the prepared product is highly dependent on the droplet characteristics, interfacial composition, and the high- or low-energy processing method used [50,102,103]. Similarly, the use of vesicular systems in the beverage scale requires a balance between the stability of the system against acidity, heat and storage stress and the release of the drug during digestion. This has been mentioned previously in the context of the broader review [29] for coated liposomal systems.

Hybrid carrier design is the most promising general solution, as it spreads the solubility enhancement, taste masking, oxidative protection, and release control among multiple structural elements, rather than requiring one carrier to meet all the criteria [115]. This is shown by whey protein isolate–β-cyclodextrin nanocomplexes that simultaneously stabilize EGCG and mask its bitterness in beverage systems (detailed in Section 3.3.2) [109]. Other evidence from emulsion and Pickering studies indicates that antioxidant-rich particles at the interface, antioxidant-rich natural stabilizers, or antioxidant-rich reservoirs in the form of liposomes may also have the potential to enhance the oxidative stability without compromising the carrier function, but there are practical limitations of complexity and cost for translation [104,114].

**Figure 2:**
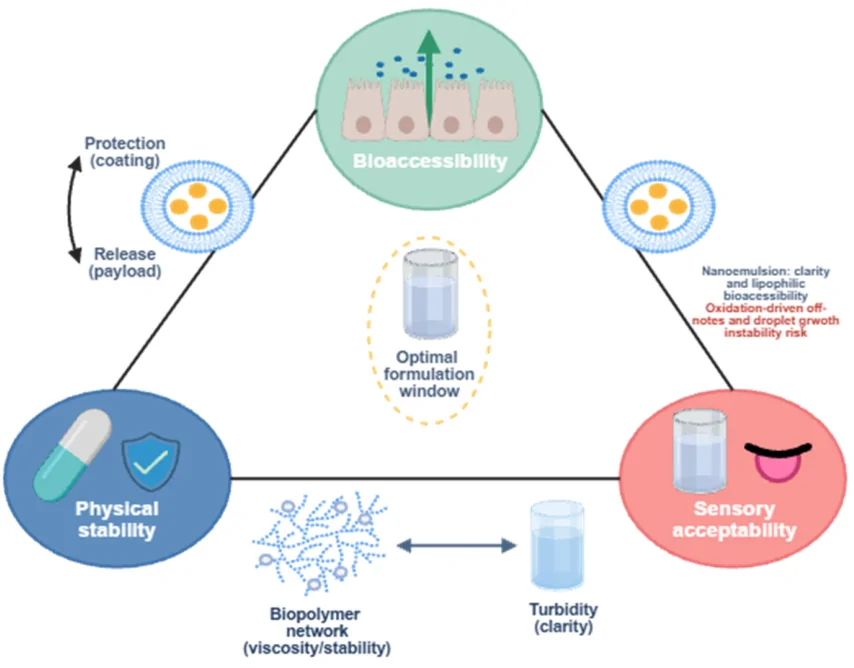
The three-way optimization problem in beverage delivery design. Functional bioaccessibility, physical stability and sensory acceptability form a trade-off space in which the viable product occupies a central window rather than maximising any single vertex. Each edge names a documented tension: coating protection versus digestion-stage release (left; [29]); clarity and lipophilic bioaccessibility carrying an oxidation penalty that manifests both as sensory off-notes and as physical instability (right; [103,104]); and network-driven viscosity/stability versus turbidity and loss of clarity (bottom; [48,106]). Vertex and edge placements are qualitative syntheses of Section 1.2, Section 2.2 and Section 3.2, not measured coordinates.

The most promising beverage candidates are therefore not necessarily the systems that maximise nominal encapsulation, but those that remain stable during processing and storage and retain sensory palatability [50,104]. Figure 2 shows these trade-offs between pairs of variables, as first described in Section 1.2.

### 3.3. Advanced Strategies for Flavor-Preservation and Masking in Encapsulated Matrices

These sensory compromises can be further exploited in advanced encapsulation to overcome flavor instability and off-flavors that hinder the acceptability of fortified beverages. In beverage systems, these strategies are most applicable to three formulation problems: protection of volatile aroma compounds during processing and storage, masking of bitterness or astringency of bioactive ingredients, and controlling release to minimize undesirable taste during oral consumption while maintaining functionality after ingestion [116,117,118].

#### 3.3.1. Protecting Labile Flavors: Mitigating Volatility and Oxidation

Preservation of flavor compounds is a major challenge in beverage formulation, as they are often volatile and sensitive to heat, light, pH, and oxidation [117,119]. The use of encapsulation technology enhances flavor retention by minimizing volatile losses through evaporation and chemical degradation during processing and storage by creating a physical barrier between the food product and the surrounding environment [119,120]. In beverages, in particular, flavorings are often prepared as microemulsions or nanoemulsions to maintain the quality of the flavor and to avoid the appearance of undesirable flavor notes [116,117].

Nanoencapsulation is especially beneficial for citrus flavors that are sensitive to oxygen. Emulsion-based systems have been reported to increase the stability of oxygen-sensitive flavor substances and antioxidants can be added to these systems to slow down degradation [103,120]. Citral is a relevant beverage example as it undergoes degradation by acid-catalyzed and oxidative reactions, and β-carotene and black tea extracts inhibited citral degradation during 60 days of storage, though degradation was accelerated at 50 °C [120]. Similarly, d-limonene is a key citrus flavor component that is used in food and beverages, but its low water solubility, volatility, and susceptibility to oxidation make it difficult to use in beverages, so nanocarriers such as nanoemulsions, nanoliposomes, and related nanocarriers are being developed to protect it [121,122]. One d-limonene nanoemulsion study showed that the formulations were physically stable in the pH range 3–9 and 0–500 mM NaCl, and 91% of the d-limonene remained after 30 days at 5 °C, directly relevant to acidic and ion-containing beverage matrices [122]. Lipid based carriers are also suitable for beverage aroma control; an encapsulated EO using liposome–alginate carrier was able to reduce the amount of cinnamaldehyde in the headspace of an acidic beverage by approximately 20% and enhance the masking of the odor in sensory testing [123]. Another aroma compound of interest to beverages, linalool, was also found to be unstable in beverages, and a food-compatible lipid vesicle system was found to enhance its stability over a range of pH, temperature, and ionic strength and allow for controlled release, though this was not in a specific beverage trial, but rather a general functional food system [124].

#### 3.3.2. Masking Unpleasant Tastes: Cyclodextrin Complexation and Complementary Encapsulation

The most well-documented method of bitterness masking provided in the literature is cyclodextrin inclusion. The hydrophobic cavity and hydrophilic exterior of cyclodextrins allow them to bind hydrophobic flavor or bioactive molecules by hydrophobic interactions, thereby enhancing the stability and controlled release of the molecules in aqueous systems [120]. Cyclodextrin inclusion should therefore be regarded as a well-established, leading strategy rather than an unequivocal “gold standard”.

The most compelling example is EGCG, which is found in tea. EGCG adds bitterness and astringency to tea drinks and is also light, heat, and oxygen sensitive [109,125]. The encapsulation efficiency of the nanocomplexes was 79.61%, the bitter and astringent taste was masked in sensory testing, and this was thought to be due to the bitter moiety being inserted into the hydrophobic cavity of β-cyclodextrin [109]. These nanocomplexes also exhibited sustained release in simulated gastrointestinal conditions and were suggested for tea beverages and related products [109]. Additionally, β-cyclodextrin has been demonstrated to effectively reduce the bitterness of green tea extract and to safeguard the active ingredients from heat, acid/base and time stress [126]. Another relevant comparator is the lipid-based nanovesicles: phospholipid nanovesicles containing EGCG achieved 92.1% efficiency and decreased the bitterness by 1/12th of the unencapsulated control. Concurrently, γ-cyclodextrin enhanced the adsorption of EGCG to phospholipids and facilitated sustained release [127].

The general relevance of cyclodextrins to the beverage industry is also reinforced. In foods, the term “encapsulation” is used to mask odors and tastes in general [118,128]. Specifically, the complexation of cyclodextrins with the extracts resulted in a decrease in the bitter taste in various herbal tinctures, and the efficacy of the complexation varied depending on the type of cyclodextrin and the extract; β-cyclodextrin was most effective in the case of marrubium, as it formed an inclusion complex with marrubiin [129].

#### 3.3.3. Controlling Flavor Release: Triggered Barriers for Oral Bypass

The evidence is in favor of adding triggered release, but with some caution. In beverages, the encapsulation systems are now designed to protect the product, but also to release the product in a controlled, sustained, burst or targeted manner [116,118,119]. In general, flavor encapsulation systems can release the flavor in a controlled and sustained manner, and the release rate can be influenced by the structure of the carrier and the physicochemical characteristics of the flavor [120].

Coffee containing milk beverages are the best example for beverage-relevant evidence. The caramel flavor microcapsules exhibited good release in simulated intestinal fluid and poor release in simulated gastric fluid, and their addition prolonged the flavor retention in the storage process [119,120]. Oral to intestinal targeting is better mechanistically supported by pH responsive biopolymer particles. The encapsulated fucoxanthin was released from calcium alginate hydrogel beads less than 3.78% in simulated gastric fluid and more than 99% in simulated intestinal fluid [130]. Alginate/carboxymethyl chitosan/gelatin hydrogel beads also exhibited sustained release, releasing 39% in gastric fluid within 2 h and releasing gradually in intestinal fluid [131]. The chitosan–alginate polyionic particles are also pH sensitive and interact with intestinal mucus, which makes them suitable for use as responsive oral carriers [132]. A more recent fully polysaccharide system for curcumin showed that it released less than 20% in simulated gastric conditions and then released completely in the intestine, corroborating the overall idea of oral bypass and intestinal delivery [133].

## 4. A Decision Framework for Beverage Delivery System Selection

### 4.1. The Adaptation of Solid-Food Encapsulation Logic to Aqueous, Acidic Environments

The transfer of encapsulation systems from solid matrices to acidic beverages adds physicochemical stressors which are not present in solid or neutral-pH formats. Non-dairy matrices generally have lower pH and buffering capacity than dairy matrices, which means that the encapsulated bioactives are subjected to higher acid stress, leading to increased proton diffusion across biological membranes and affecting cellular metabolism [134]. The acidic beverage condition (pH ≤ 4.5) causes the encapsulated probiotics to use up their energy surplus for maintaining intracellular pH, leading to cell death [135], while the most destructive effect on alginate capsules is observed in acidic conditions, with losses up to 47.4% after 30 min [136].

Moisture ingress adds to this challenge. In aqueous beverage environments, water acts as a strong plasticizer for spray-dried polysaccharide wall materials (such as maltodextrin), thereby depressing their glass transition temperature (*T_g_*) and converting glassy protective shells into permeable rubbery matrices [137,138,139]. This hydration-induced matrix softening accelerates the premature diffusion and degradation of encapsulated phytochemicals under pasteurization and storage conditions [137,138]. Furthermore, the ionic strength of beverage matrices promotes sub-micron particle aggregation (<1 µm) and leads to sedimentation or mouthfeel defects for larger capsules (>100 µm) [139], while capsule swelling can disrupt the protective polymer network [140].

Although there are these problems, there is a general consensus that microencapsulation can be transferred to acidic beverage matrices. The vitamin C loss in apple juice was 0.57% after 24 days of storage with yeast-encapsulated ascorbic acid compared to 15.1% loss in the unencapsulated ascorbic acid [141]. Microencapsulation using jackfruit seed flour improved the retention of *β*β-carotene in acidic apple juice (pH~3; detailed under thermal processing in Section 4.3) [142]. The retention of anthocyanin in model beverages was 97.6% in FOS–whey protein microcapsules at 4 °C, compared to 68.8% for free anthocyanins after eight days [143]. In Iranian Doogh (pH~4.5), calcium alginate microcapsules coated with chitosan and Eudragit S100 preserved the viability of L. acidophilus for 42 days without any acid and metabolite accumulation [144], and in coconut water extract, symbiotic microcapsules preserved the viability of L. acidophilus above 6 log colony-forming units (CFU)/mL for 42 days [145].

Protein–polysaccharide complexes are a very promising approach for acidic systems. These complexes are generated by electrostatic, hydrogen bonding, and hydrophobic interactions that create dense internal networks that protect bioactives from environmental stress [146]. The electrostatic repulsion caused by polysaccharide adsorption onto protein surfaces prevents secondary aggregation destabilizing uncomplexed proteins near the isoelectric point [147], and Maillard-conjugated complexes provide protection over the pH range of 3–7. Soybean soluble polysaccharide–whey protein complexes exhibited excellent colloidal stability in acidified whey protein beverages [148], and casein–alginate conjugates showed improved stability of curcumin, with a bioaccessibility of 57% compared to less than 10% for free curcumin [149]. But the choice of wall material is crucial: gum Arabic–pea protein complexes did not stabilize emulsions at pH 4.5, although they did at pH 8.0 [150], and reviews warn that the encapsulation efficiency, water solubility, particle size, and release rate should all be tested for each new beverage matrix [151].

### 4.2. Comparative Decision-Support Framework: Compatibility, Stability, Scalability, and Cost

Several models have been suggested for the systematization of delivery system selection. The most explicit quantitative model is a multi-criteria decision analysis (MCDA) that combines four dimensions of evaluation: technical performance, economic viability, regulatory compliance and environmental sustainability [139]. For example, in a cited multi-criteria decision analysis (MCDA) evaluating omega-3 beverage fortification [139], spray drying achieved the highest overall suitability score (8.03) due to low processing cost and high industrial throughput; complex coacervation achieved superior encapsulation efficiency but ranked intermediate (7.41) due to material costs and regulatory constraints, while liposomal encapsulation received the lowest overall score (6.05) due to vesicular fragility and formulation expense [139]. Flexibility of the framework can be exploited for shifting the weight, for example, to give more importance to technical performance (50%) than economics (20%) in premium products would favor coacervation or liposomal systems [139].

This multi-dimensional logic is reinforced by complementary frameworks. The Delivery by Design (DbD) approach focuses on the design of each system for both the encapsulated compound and the target food matrix, systematically considering the colloidal systems with respect to particle composition, size, morphology, stability, optical properties, rheology, and sensory attributes [152]. Design-driven matching principles suggest that hydrophobic or oxidation-sensitive bioactives are more suitable for nanoemulsions or multilayer interfacial systems, whereas hydrophilic compounds and probiotics are better suited for hydrogels or protein–polysaccharide networks; and small, low-viscosity systems are better suited to liquid products [153]. In the industrial sector, matrix compatibility and sensory impact are still important challenges because delivery systems are often not completely compatible with the end product, are too expensive, or are not accepted by the consumer [154].

The cost and scalability constraints are very strict. Industrial formulation constraints usually require that delivery systems add only marginal cost per finished beverage unit, restricting high-cost carrier systems to premium categories [139]. Furthermore, scaling from bench-scale to continuous pilot extrusion frequently widens particle size distributions, increasing batch-to-batch variability and the risk of nozzle clogging or sedimentation [139,155]. The most scalable method is spray drying which can partially degrade heat sensitive compounds during capsule formation, and there is a trade-off between cost and functional preservation [20].

Nanoemulsion technology has gained widespread commercial traction in functional waters, teas, and clear sports drinks due to its ability to encapsulate lipophilic bioactives without causing light scattering or visible turbidity [50,115]. Nanoemulsions are superior to liposomes for hydrophobic bioactives; droplets of less than 50 nm yield optically transparent dispersions for clear beverages, and bioavailability of curcumin from nanoemulsions was nine times greater than from the crystalline form [156]. The advantage of liposomes is that they can be used to simultaneously encapsulate hydrophilic and hydrophobic compounds because of their amphiphilic bilayer structure [64]. Since none of the technologies is ideal for all four MCDA dimensions, research is now focused on hybrid systems, such as the integration of nanostructured lipid carriers into spray-dried microcapsule matrices to achieve the best of both worlds: the nanoparticle bioavailability enhancement and the spray-drying scalability and regulatory acceptability [139,157]. Each carrier system is then correlated to its target beverage matrix, optimal payload, processing-resilience profile and primary industrial bottleneck in Table 2, which serves as a comparative qualitative decision matrix for beverage developers to guide formulation design.

**Table 2:** Comparative Decision-Support Matrix: Matching Carrier Architectures to Beverage Matrix, Processing, and Payload Constraints.

Carrier System	Target Beverage Matrix	Bioactive Payload	Processing Resilience	Main Constraint	References
**Nanoemulsions**	Clear/translucent fortified waters and juices (requires droplets <200 nm to minimize light scattering).	Lipophilic actives (CoQ10, carotenoids, omega-3s) and oxygen-sensitive volatile flavors (citral, d-limonene).	Thermodynamically unstable; suppress droplet growth by reducing Laplace pressure. High-energy fabrication required. High interfacial area accelerates lipid oxidation (pentanal/hexanal off-notes) unless antioxidant-loaded.	Managing interfacial lipid oxidation and off-note generation over extended storage; thermodynamic instability leading to creaming, sedimentation, or Ostwald ripening.	[41,50,103,104]
**Pickering Emulsions**	Opaque, creamy, or reduced-fat formulations, including plant-based dairy cream alternatives. Inherently cause turbidity due to gel-like particle networks.	Lipophilic ingredients; fat reduction via W/O/W multiple emulsions.	High mechanical and thermal stability; solid food-grade particles (starch, cellulose, chitosan) form dense, irreversibly adsorbed interfacial barriers that suppress coalescence and retard Ostwald ripening. Robust against pH, temperature, and ionic strength fluctuations.	Incompatibility with clear beverage formats; increasing particle content to improve creaminess can reduce lubricity, increase brittleness, and introduce grittiness or textural defects.	[43,44,53,106,158]
**Vesicular Systems (Liposomes and Niosomes)**	High-value nutraceutical drinks, dairy, and juice models. Niosomes maintain clarity better and show superior pasteurization stability in orange juice compared to liposomes.	Mixed-polarity payloads (simultaneous hydrophilic and lipophilic delivery, e.g., vitamins, polyphenols, flavors) and poorly soluble bioactives.	Liposomes are fragile to oxidation, hydrolysis, and pH/thermal shifts, requiring GRAS biopolymer coatings (pectin, alginate) for reinforcement. Niosomes (non-ionic surfactants) offer superior storage/pasteurization stability and outperform liposomes in palatability.	Liposomal payload leakage, oxidation, and cost; excessive biopolymer stabilization can impede intestinal micellization. Niosomes are constrained by a narrow food-grade surfactant toolbox.	[29,31,33,36]
**Protein–Polysaccharide Complexes**	Acidic beverages (pH 3.0–4.5) where isoelectric precipitation is a risk; validated in tea and fermented whey systems.	Hydrophilic polyphenols (EGCG, anthocyanins), curcumin conjugates, and probiotics.	Electrostatic networks and Maillard-conjugated complexes shield proteins near the isoelectric point, preventing secondary aggregation and delaying degradation; polyphenol–protein interactions govern stability and astringency masking.	Antisolvent and pH-driven scale-up complexity; wall-material dependency; batch-to-batch variation and particle size standard deviation increases at industrial scale.	[62,148,150,159,160,161,162]
**Microbial EPS (In-Situ)**	Fermented, viscous, or gut-targeted functional drinks (e.g., fermented soy, dairy/non-dairy biothickeners).	Endogenous metabolites, prebiotics, and bioactive peptides. Acts as a natural stabilizer and prebiotic matrix.	Generated in-situ via precision fermentation, contributing rheology, physical stability, and emulsification. Resists upper GI digestion to undergo colonic fermentation (driving SCFA production). Collapses multi-step supply chains into a single bioprocessing step.	Low production yields; structure-dependent fermentability; host-to-host microbiota variability creating “non-responder” phenotypes; lack of quantitative in vivo controlled-release data for co-loaded exogenous actives.	[66,69,81,83]

**Abbreviations**: CoQ10, coenzyme Q10; EGCG, epigallocatechin gallate; W/O/W, water-in-oil-in-water; GRAS, generally recognized as safe; EPS, exopolysaccharides.

### 4.3. Processing Resilience: Surviving Pasteurization, UHT and Non-Thermal (HPP, PEF) Treatments

The three dimensions that are interconnected in the context of thermal processing of encapsulated bioactives are carrier physical stability, bioactive retention, and downstream bioavailability. A uniform threshold pattern is observed throughout the carrier types. Taxifolin loaded nanostructured lipid carriers were found to be stable at temperatures ≤ 70 °C, while the treatment of 80–90 °C increased the particle size by ~200 nm and decreased the encapsulation efficiency (EE) [163]; vitamin D_2_ nanoemulsions with particle size < 200 nm were stable in the range of 30–90 °C [16]. The type of emulsifier is critical: protein stabilized emulsions are sensitive to aggregation at temperatures above denaturation (65 °C), whereas polysaccharide based emulsions are more resistant to temperature changes [164]. The modified lecithin stabilized nanoemulsions of astaxanthin were found to be stable for 30 min at 60–120 °C while the nanoemulsions stabilized by sodium caseinate exhibited droplet growth [165].

The retention of phytochemicals is always enhanced during thermal processing with encapsulation. For apple juice (pH~3), the encapsulated β-carotene remained at 6.22 µg/mL after pasteurization at 70 °C for 15 min compared to 1.48 µg/mL for the unencapsulated controls, which is approximately fourfold higher [142]. The total antioxidant capacity of bacterial cell carriers containing muscadine grape juice polyphenols was about 93% after 60 min at 90 °C compared to 61% for free anthocyanins [166]. However, the lowest release and highest retention of encapsulated phenolics were obtained at pH 3.0, suggesting that acidic conditions further protect encapsulated phytochemicals [167]. The in vitro bioaccessibility of coenzyme Q10 was 1.8-fold higher for the nanoemulsions than for the oil-dissolved forms, and 2.8-fold higher when the nanoemulsions were added to a high-protein beverage [168], while the bioavailability of astaxanthin was 33% for modified lecithin and 6% for sodium caseinate [165]. The evidence of UHT specific protection (≥135 °C) is still limited and data on thermal stability are frequently obtained by thermogravimetric analysis instead of real UHT processing [169].

There are complementary pathways with non-thermal technologies. High-pressure processing (HPP) inactivates microorganisms at moderate temperatures without altering the physicochemical, nutritional and sensory properties [170], and does not alter covalent bonds during pressurisation, resulting in minimal alteration of food biomolecules [171]. HPP has been shown to preserve higher levels of anthocyanins, flavonoids and phenolics than thermal pasteurization in various beverage matrices [171], and in a soymilk beverage, HPP increased the bioaccessibility of isoflavones by 47.32% compared to thermal processing [172]. HPP, when applied in combination with encapsulation, retained 25–35% higher phenolics and carotenoids in avocado puree than the controls during 22 days of refrigerated storage [173]. Adoption rates, however, are low, at 18% of large-scale producers, and the processing costs are higher than thermal pasteurization, at about seven times higher, and polyphenol oxidase inactivation is lower, at 15–38% [174,175,176].

Pulsed electric field (PEF) processing creates electroporation in cell membranes, which opens them up and makes it easier to introduce desirable components [177]. PEF also polarizes protein molecules in an electric field, exposing hydrophobic groups that adsorb at the surface of oil droplets, thereby decreasing the particle size at the oil–water interface [178,179]. The emulsion stability (ES) of milk protein concentrate treated with PEF was 70.2% higher than the untreated sample [180], while whey protein isolate had a 250% increase in emulsion stability index (ESI) compared to the untreated sample [181]. PEF at 200 Hz/168.4 kJ/L resulted in a 79% and 77% increase in total phenolic content and vitamin C, respectively, of raspberry juice [177], and a 35.40% increase in the bioaccessibility of isoflavones in soymilk [172]. PEF effects are dose-dependent: low to moderate PEF field strengths are beneficial, while prolonged exposure leads to hydrophobic and covalent interactions that result in aggregates that may offset the stabilization effect [182]. Challenges to industrial implementation include electrode corrosion, non-uniform field distribution, and regulatory requirements for GRAS status or novel food classification [175,176].

## 5. Sustainability, Regulation, and Market Translation

The commercial translation of advanced delivery systems is influenced by the distinct physicochemical environments and consumer expectations of different beverage categories [1,3]. In practical formulation, these systems are usually represented across three functional liquid categories: (i) clear ready-to-drink (RTD) waters and fortified teas, which usually rely on sub-200 nm nanoemulsions to disperse lipophilic vitamins and volatile flavors without causing visible turbidity [1,50]; (ii) acidic fruit juices and beverage systems (pH ≤ 4.5), where biopolymer complexes and microcapsules are frequently investigated to protect labile phytochemicals against thermal pasteurization and acid-induced precipitation [141,142]; and (iii) plant-based dairy alternatives and fermented beverages, where Pickering emulsions and microbial exopolysaccharides (EPS) are actively explored for fat-mimetic mouthfeel, physical stabilization, and prebiotic positioning [52,66]. Translating these diverse carrier formats from bench-scale prototypes to commercial scale involves balancing environmental life-cycle sustainability against processing energy demands (Section 5.1), properly aligning with divergent international regulatory frameworks (Section 5.2), and addressing transformation-dependent safety data gaps in dynamic liquid matrices (Section 5.3).

### 5.1. Green Carrier Selection and Life-Cycle Sustainability Assessment

In published multi-criteria decision analysis (MCDA) models [139], technical performance and economic viability are combined to evaluate carrier suitability; however, rankings based strictly on bench-scale stability can fail to consider the product’s full life-cycle environmental footprint. Bio-based, biodegradable or clean label ingredients are not necessarily environmentally friendly. Operational life-cycle metrics should therefore be assessed in nine interrelated burden categories: renewable feedstock selection, agricultural land and water demand, extraction solvent burden, processing intensity, electrical energy consumption, packaging requirements, material biodegradability, end-of-life waste behavior, and net greenhouse-gas (GHG) emissions [115,183,184]. Agricultural cultivation is resource-intensive, requiring substantial arable land and irrigation water, while chemical fractionation often requires organic solvents or thermal processing. The ecological advantages of renewable sourcing can be partly counterbalanced by the electrical energy needed for nanoscale mechanical fabrication, which is the energy hotspot in the case of high-pressure homogenization and microfluidization [115,184].

These life-cycle dimensions when applied to carrier classes demonstrate real formulation compromises. Virgin plant polysaccharides (e.g., starch, alginate, carrageenan used as spray-drying carriers [185]) are biocompatible, but also have significant burdens in terms of agricultural land use and processing intensity, and in food-ingredient processing, thermal drying can dominate operational environmental burdens across several life-cycle impact categories [184,185]. An alternative to primary agricultural burdens is to use upcycling of agri-industrial side streams like citrus pomace, tea residues, or cheese whey, which is a circular-bioeconomy approach [185]. However, industrial translation requires the ability to control the compositional variability of batches without relying on energy-intensive washing, enzymatic conditioning, or chemical cross-linking, which may reduce the net environmental benefits (detailed in Section 4.1) [136,140].

The alternative of precision fermentation to produce microbial exopolysaccharides (EPS) is a developing technology that avoids the use of agricultural land and extraction costs, and yields functional hydrocolloids in situ from simple feedstocks [66,81]. However, the use of these is limited by low batch yields, separation energy downstream, structure-dependent fermentability by host microbiota, and few in vivo kinetic release data during liquid-matrix gastrointestinal transit [69,71]. Submicron emulsions, which are inherently vulnerable to lipid oxidation, often require high-barrier packaging or refrigerated cold-chain distribution; this packaging and storage burden can substantially elevate operational greenhouse-gas emissions compared to ambient-stable biopolymer complexes [103,104,156].

Environmental fate at post-consumption end-of-life is different for the various waste fractions: the water-soluble biopolymer fraction in beverage rinses is easily biodegraded in municipal wastewater treatment plants, while solid carrier residues and packaging waste in landfills are slowly degraded under anaerobic conditions, with the potential to release methane [183,184]. Due to the limited number of quantitative cradle-to-gate LCA data available in the literature for food-grade nanocarriers, environmental benignity should be systematically assessed for these distinct burden categories, and not taken for granted based on the origin of the feedstocks [115,184].

### 5.2. Nanotechnology Classifications and Regulatory Pathways Across Key Jurisdictions

Navigating international markets is a mix of conflicting jurisdictions and safety data expectations. The European Union has a structured, legally binding risk-assessment framework, based on the European Commission’s updated Recommendation on the definition of nanomaterial (2022/C 229/01) [186]. It defines a nanomaterial as one in which 50% or more of particles in the number-based size distribution have one or more dimensions in the range of 1 nm to 100 nm [186]. Engineered nanomaterials require pre-market novel food authorization under Regulation (EU) 2015/2283 [187], labeled as ingredients (with the addition of the word “(nano)” to the ingredient name) under Regulation (EU) No 1169/2011, and undergo tiered toxicological testing [187,188,189]. In its guidance on technical requirements for small particles [190,191], EFSA recommends nanospecific risk assessment for food ingredients containing sub-500 nm particles that are insoluble under simulated physiological conditions. On the other hand, if gastrointestinal dissolution is rapid and complete, it can serve as a basis to bypass extensive nanospecific testing for digestible organic carriers, making dissolution screening a filter rather than a barrier [190,191].

The United States and the United Kingdom assess nano-enabled ingredients mainly in a product-specific and functional manner in existing food safety legislation, as opposed to the EU approach of categorization. The United States Food and Drug Administration (FDA) provides non-binding guidance for industry to consider whether an engineered product that involves nanotechnology (1–100 nm or up to 1000 nm exhibiting dimension-dependent properties) [192], evaluating safety on a case-by-case basis under existing Generally Recognized As Safe (GRAS) or Food Additive pathways [192,193]. Formulators should consider revisiting traditional scale GRAS conclusions since particle-size reduction can affect the biological behavior [192,193]. In the post-Brexit UK, novel foods are regulated by the Food Standards Agency (FSA) and Food Standards Scotland (FSS) with scientific advice from the Committee on Toxicity (COT) based on risk assessment principles that take into account exposure and matrix context and do not rely on strict statutory categorization of nano [194,195]. As a result, delivery systems that secure rapid market entry in the United States through self-affirmed GRAS may encounter a data-intensive, multi-year novel food authorization process in the European Union [192,194].

These jurisdictional details are complicated by analytical considerations in liquid beverage matrices. Traditional regulatory exposure metrics focus on mass concentration instead of particle number, and validated reference standards for complex, low-viscosity liquid matrices are limited [196,197,198,199]. However, in commercial beverages, it is technically challenging to differentiate between engineered nanocarriers and native colloidal assemblies, such as proteins, lipid droplets, and soluble fibers, because common hydrodynamic sizing techniques can provide different size distributions in turbid or acidic beverages [200,201]. For formulators, in vitro gastrointestinal fate and physiological stability are more critical than initial nominal dimensions in determining toxicological data requirements, because rapid degradation can bypass extensive nanospecific testing even when nominal particle size triggers statutory categorization [197,201]. The early-stage formulation screening aligned with European dissolution and degradation criteria is therefore a pragmatic, evidence-based approach to support safety in global markets [191,201].

### 5.3. Critical Safety Data Gaps in Liquid-Matrix Encapsulation

The safety evidence base to populate dossiers for next-generation beverage encapsulants is still limited by the specific physics and chemistry of liquid matrices [188,189]. Rapid particle dispersion is required for high-speed packaging lines, while high ionic strength in beverage formulations will promote sub-micron particle aggregation, and capsules > 100 µm will lead to sedimentation or mouthfeel defects [139]. As detailed in Section 4.1, acidic beverage matrices (pH ≤ 4.5) can promote hydrogel swelling and accelerate viability losses in unbuffered probiotic microcapsules [135,136,140]. While multi-layer biopolymer shells have been shown to help sustain probiotic survival during acidic storage [144], applying dense coatings to lipophilic bioactives can introduce a mechanistic trade-off: as observed with pectin-coated liposomes in Section 2.1, excessive interfacial stabilization may restrict bile-salt and lipase access, thereby reducing intestinal bioaccessibility [29]. Therefore, the ingested state of the materials cannot be characterized at the bench scale, and toxicological risk assessments must account for the effective dimensions and structural changes at the intestinal absorption site [197].

Low-viscosity continuous liquid phases offer little matrix protection, meaning that the digestibility of the carrier biopolymer directly influences the release rate of the payload, the stability of the interface, and the interaction with the epithelial cells [197,202]. The intestinal β-carotene bioaccessibility of digestible zein nanospheres is 37 ± 1% under static gastrointestinal digestion (INFOGEST) with pancreatin, while indigestible ethylcellulose nanospheres only reach 8.3 ± 0.1% [202]. In addition, the uptake of nanoemulsion droplets into Caco-2 monolayers is optimal at 120 nm, while larger droplets (380 nm) disrupt the integrity of the epithelial barrier [203]. In addition, the large surface area of beverage nanoemulsions causes interfacial lipid oxidation, which produces volatile off-flavor aldehydes (pentanal and hexanal) [103,104,113]; however, appropriate emulsifier selection (such as modified lecithin rather than sodium caseinate) has been observed to reduce heat-induced degradation and favor downstream bioaccessibility (Section 4.3 [165]). Importantly, the effects of persistent inorganic additives (TiO_2_, SiO_2_, ZnO, Ag) differ markedly from digestible organic biopolymers [197]: In vivo Gallus gallus models show that food-grade TiO_2_ and ZnO reduce the surface area of the villi in the duodenum, alter the depth of the crypts, and upregulate pro-inflammatory cytokines (tumor necrosis factor-α (TNF-α), nuclear factor-κB (NF-κB), and interleukin-8 (IL-8)), while Ag nanoparticles remain cytotoxic and genotoxic despite digestive biotransformations [204].

Low-viscosity drinks pass through the stomach quickly, allowing microbiome interaction to be a chronic-exposure situation, while solid foods undergo slow gastric breakdown, delivering encapsulants and exopolysaccharide (EPS) matrices directly to the colon every day [69,71]. Lactic acid bacteria-derived EPS are generally not destroyed by upper gastrointestinal transit, but rather undergo fermentation in the colon, resulting in an increase in alpha diversity and production of short-chain fatty acids (SCFA) such as butyrate and propionate [71,81]; however, fermentability is highly structure-dependent and host-to-host microbiota variability leads to the emergence of distinct “non-responder” phenotypes [69,83]. The liquid dynamics of these beverages are poorly represented by standard preclinical testing models, such as static Caco-2 monolayers and standard INFOGEST protocols, which lack fluid shear stress, protective mucus dynamics, and rapid gastric emptying [16,94,97,203]. To address these transformation-dependent safety questions, emerging microphysiological gut-on-a-chip platforms may serve as promising pre-screening tools to help separate carrier degradation products, payload exposure, and matrix effects under physiological flow [83,205].

## 6. Forward-Looking Perspectives: Precision Delivery and AI-Driven Formulation

Next generation nutraceutical beverages must go beyond static in vitro assays to be successfully translated. Beverage carriers exhibit very different behavior in low-viscosity, high-transit liquid matrices than solid foods, and existing static digestion models coupled with Caco-2 Transwell assays do not account for fluid dynamics, shear stress, and mucus interactions that govern in vivo bioavailability. An emerging forward-looking perspective combines microphysiological gut-on-a-chip and organoid models with computational AI tools, not as replacements for human clinical studies, but as exploratory pre-screening methods to help prioritize formulation candidates and find potential matrix or sensory liabilities prior to pilot-scale trials.

### 6.1. Gut-on-a-Chip and Organoid Platforms for Exploratory Screening in Liquid Matrices

Static 2D monolayers lack the luminal flow, cyclic strain, and oxygen gradients that are disproportionately affecting gastrointestinal fate of liquid matrices. Dynamic microphysiological systems (gut-on-a-chip platforms and 3D intestinal organoids) overcome these limitations by maintaining villus–crypt organization, allowing multicellular or microbiota co-cultures, and recreating flow-dependent permeability that better mimics ex vivo and in vivo behavior than Transwell formats [9,206,207,208,209].

The most relevant microphysiological platforms for translation are the ones that have been tested in liquid food and beverage matrices and not only in idealized drug solutions. A key proof-of-principle is the compartmentalized gut-on-a-chip coupled to online mass spectrometry (MS) readouts, which enables digestion, an intestinal epithelial barrier, and time-resolved detection of transport to be performed in a single digestion-to-absorption workflow [210,211]. This system was tested with a pharmaceutical model probe compound (verapamil), but showed that the transport kinetics were measurably affected by the presence of constituents in the beverage matrix, as compared to aqueous buffer controls, indicating that the presence of constituents in the beverage matrix actively affects mucosal permeability. Table 3 summarizes representative proof-of-concept studies that have been evaluated on microphysiological platforms in liquid food and beverage matrices to map each platform to the matrix evaluated and the experimental outcome.

Together, these systems shift the main question from ‘does the carrier protect the payload?’ to ‘what actually survives digestion and reaches the gut lining in a real liquid matrix?’, a question that traditional static assays cannot answer. Given the current constraints around fabrication costs, throughput, and regulatory standardization, these platforms can be used as early-stage filters to help prioritize promising formulations, rather than as replacements for human clinical studies [9,209,212].

**Table 3:** Microphysiological platforms and proof-of-concept studies in liquid food matrices.

Platform	Beverage Matrix	Experimental Outcomes and Relevance	References
**Compartmentalized Gut-on-a-Chip**	Apple Juice (Liquid Matrix)	Outcome: Verapamil (Model probe compound) uptake was measurably slower in apple juice than in aqueous buffer. Relevance: Illustrates that liquid matrix constituents can influence mucosal transport kinetics, thereby demonstrating proof-of-concept for integrated chip-based screening workflows.	[210,211]
**Digestion-on-a-Chip**	Lactoferrin/Casein (Dairy Proteins)	Outcome: Continuous-flow digested lactoferrin faster than batch methods; downstream Caco-2/HT29-MTX co-cultures successfully formed villi-like epithelium with ex vivo-like permeability. Relevance: Validates matrix-specific digestion speeds and barrier formation for protein-fortified drinks.	[213,214]
**Dynamic 3D Gut Models**	Curcumin Nanoemulsions/Carotenoids	Outcome: Curcumin nanoemulsions yielded higher permeability rates that closely matched in vivo trends. Relevance: Confirms that dynamic flow and mucus gradients accurately predict the intestinal fate of lipid-based liquid delivery systems.	[203,209]
**Microfluidic Shear-Force Model**	Complex Oligosaccharides (Nutritional Glycans)	Outcome: Intestinal peristaltic shear force fundamentally altered how human milk oligosaccharides (hMOs) modulate tight junctions (ZO-1) and enhanced the adhesion of the commensal bacterium *Lactobacillus plantarum* WCFS1 to the epithelium. Relevance: Demonstrates that dynamic fluid flow enhances glycan-mediated barrier integrity and probiotic adhesion, capturing beneficial shear-dependent interactions that occur in the living gut.	[215]
**Gut-on-a-Chip and Colon Organoids**	Emulsified Liquid Matrices (Beverage Emulsifiers)	Outcome: Revealed that common food emulsifiers directly impair intestinal epithelial barrier integrity and trigger pro-inflammatory pathways and immunoglobulin E (IgE) production. Relevance: Proves that microphysiological systems can evaluate the safety and barrier-disruption risks of the structural stabilizers used in beverage formulations.	[216]

**Abbreviations**: 3D, three-dimensional; Caco-2 and HT29-MTX, human intestinal epithelial cell lines (HT29-MTX, mucus-secreting methotrexate-adapted HT29 derivative); hMO, human milk oligosaccharide; ZO-1, zonula occludens-1 (tight junction protein 1); WCFS1, strain designation of *Lactobacillus plantarum*; IgE, immunoglobulin E.

### 6.2. AI and Multi-Omics for Predictive Formulation Design

The computational engine that is needed to interpret the heterogenous data produced by advanced physiological models is provided by AI and multi-omics. Beverage formulation requires balancing particle size, digestion kinetics, epithelial transport, metabolite formation and volatile profiles. Machine learning can be used to combine multimodal data, including gas chromatography–mass spectrometry (GC-MS), gas chromatography–ion mobility spectrometry (GC-IMS), electronic noses, electronic tongues, and sensory panels, to enhance predictive performance for flavor stability and off-notes beyond what is achieved by single-modal models [217,218,219,220].

This predictive power is already seen in real-world beverage applications. Gradient boosting models trained on more than 200 chemical properties and sensory and consumer data were able to predict beer flavor profiles and consumer appreciation, and SHAP (SHapley Additive exPlanations) analysis was used to identify trace volatiles that have disproportionate sensory impact for targeted beer formulation [217,221]. Likewise, neural networks and XGBoost models have correlated flavoromics with aroma quality grades for sauce- and Jiang-flavor baijiu, identifying important aroma-active compounds to aid in spiking and threshold validation [222,223]. Platforms such as FlavorMiner can be used for molecular flavor mining to predict potential flavor notes and to identify off-flavors in complex matrices like cocoa [224].

AI is essential for predicting matrix-dependent bioavailability, in addition to sensory profiling. Artificial neural networks (ANN) trained with more than 250 molecular properties have shown to be superior to Support Vector Machines (SVM) and traditional Biopharmaceutics Classification System (BCS) to predict the food-matrix effect on oral bioavailability [225]. This is particularly important for nutraceutical beverages, where the bioaccessibility of β-carotene, resveratrol and gypenosides is significantly different in liquid matrices such as coffee, yogurt and juices [23]. At the same time, multi-omics integration, which integrates peptidomics, metabolomics, metagenomics, and molecular docking with machine learning, is being used in dairy systems to predict the release of bioactive peptides and interactions between the gut microbiota [212]. This offers a direct analytical blueprint to assess future fermented EPS beverages and hybrid nanoemulsions, moving the industry from trial-and-error formulation to precision design that is predictive and human-relevant.

## 7. Conclusions

Empirical studies suggest that advancing functional beverages beyond simple fortification benefits from matrix-aware delivery engineering. While experimental findings show that nanocarriers, including nanoemulsions, Pickering systems, and biopolymer complexes, can improve bioactive retention against thermal degradation and storage loss, physical retention alone does not necessarily equate to biological efficacy. In vitro and ex vivo observations suggest that high encapsulation retention can sometimes present trade-offs with speciated bioaccessibility or sensory palatability, highlighting the importance of evaluating carriers against both digestive release and organoleptic attributes.

Synthesizing these findings across processing, sustainability, and regulatory literature indicates that commercial translation involves balancing several operational considerations. Beyond formulation stability, delivery systems face diverse industrial processing regimes (such as thermal pasteurization, UHT, HPP, and PEF) while presenting life-cycle trade-offs across fabrication energy demands and end-of-life waste stream. On the other hand, comparative regulatory analysis suggests that market translation is aided by proactive alignment with jurisdictional criteria, where gastrointestinal dissolution behavior can provide a helpful scientific basis to support potential testing waivers.

To help navigate these complex considerations, we propose a conceptual matrix-aware framework intended to better connect bench-scale observations with translational realities. Within this perspective, emerging microphysiological platforms (such as gut-on-a-chip and intestinal organoids) and AI-assisted multi-omics are positioned not as replacements for human clinical trials, but as exploratory pre-screening tools that can help prioritize and de-risk candidate formulations. Ultimately, this review proposes a broader, matrix-aware framework for evaluating beverage delivery systems, aiming to balance physical protection with consumer palatability, life-cycle sustainability, and regulatory alignment.

## Figures and Tables

**Table 1:** Major beverage bioactives and their primary formulation constraints.

Bioactive Class	Beverage Application	Formulation Constraint	References
**Polyphenols**	Antioxidant and anti-inflammatory positioning (e.g., EGCG, curcumin, anthocyanins, resveratrol).	Poor aqueous solubility, astringency/bitterness, and severe digestion-stage transformation.	[1,5,14]
**Vitamins**	Deficiency prevention and fortification (e.g., vitamin C, vitamin D2).	High sensitivity to heat, pH shifts, light, and oxidative degradation during storage and pasteurization.	[15,16]
**Probiotics and Prebiotics**	Gut-health modulation (e.g., *L. acidophilus*, EPS, FOS).	Loss of viability in acidic, low-pH aqueous environments and high-osmotic-pressure matrices.	[9,11]
**Lipophilic Actives and Carotenoids**	Omega-3s, CoQ10, β-carotene, astaxanthin, fucoxanthin.	Inability to disperse in aqueous phases without causing turbidity, creaming, or lipid-oxidation off-notes.	[6,15,17]
**Flavors and Volatiles**	Sensory acceptance and aroma stability (e.g., citral, d-limonene, essential oils).	High volatility, oxidation sensitivity, and competitive displacement from carriers in complex matrices.	[18,19]
**Bioactive Peptides**	Fermentation-derived functional ingredients.	Bitterness generation and susceptibility to gastrointestinal protease degradation.	[4,20]

**Abbreviations**: EGCG, epigallocatechin gallate; CoQ10, coenzyme Q10; FOS, fructo-oligosaccharides; EPS, exopolysaccharides.

## Data Availability

This article is a review of existing published literature. No new data were generated or analyzed in this study. All data discussed in this review are available within the cited references.
